# Metagenomic Investigation of Plasma in Individuals with ME/CFS Highlights the Importance of Technical Controls to Elucidate Contamination and Batch Effects

**DOI:** 10.1371/journal.pone.0165691

**Published:** 2016-11-02

**Authors:** Ruth R. Miller, Miguel Uyaguari-Diaz, Mark N. McCabe, Vincent Montoya, Jennifer L. Gardy, Shoshana Parker, Theodore Steiner, William Hsiao, Matthew J. Nesbitt, Patrick Tang, David M. Patrick, for the CCD Study Group

**Affiliations:** 1 School of Population and Public Health, University of British Columbia, Vancouver, British Columbia, Canada; 2 British Columbia Centre for Disease Control, Vancouver, British Columbia, Canada; 3 Centre for Health Evaluation and Outcome Sciences, Vancouver, British Columbia, Canada; 4 Department of Medicine, University of British Columbia, Vancouver, British Columbia, Canada; 5 British Columbia Public Health Microbiology and Reference Laboratory, Vancouver, British Columbia, Canada; 6 Department of Pathology and Laboratory Medicine, University of British Columbia, Vancouver, British Columbia, Canada; 7 Coastal Genomics Inc., Burnaby, British Columbia, Canada; 8 Department of Pathology, Sidra Medical and Research Center, Doha, Qatar; Centers for Disease Control and Prevention, UNITED STATES

## Abstract

Myalgic Encephalomyelitis/Chronic Fatigue Syndrome (ME/CFS) is a debilitating disease causing indefinite fatigue. ME/CFS has long been hypothesised to have an infectious cause; however, no specific infectious agent has been identified. We used metagenomics to analyse the RNA from plasma samples from 25 individuals with ME/CFS and compare their microbial content to technical controls as well as three control groups: individuals with alternatively diagnosed chronic Lyme syndrome (N = 13), systemic lupus erythematosus (N = 11), and healthy controls (N = 25). We found that the majority of sequencing reads were removed during host subtraction, thus there was very low microbial RNA content in the plasma. The effects of sample batching and contamination during sample processing proved to outweigh the effects of study group on microbial RNA content, as the few differences in bacterial or viral RNA abundance we did observe between study groups were most likely caused by contamination and batch effects. Our results highlight the importance of including negative controls in all metagenomic analyses, since there was considerable overlap between bacterial content identified in study samples and control samples. For example, Proteobacteria, Firmicutes, Actinobacteria, and Bacteriodes were found in both study samples and plasma-free negative controls. Many of the taxonomic groups we saw in our plasma-free negative control samples have previously been associated with diseases, including ME/CFS, demonstrating how incorrect conclusions may arise if controls are not used and batch effects not accounted for.

## Introduction

Myalgic Encephalomyelitis/Chronic Fatigue Syndrome (ME/CFS) is a debilitating disorder of unknown aetiology estimated to cause severe, indefinite fatigue in over 350,000 Canadians [1]. It has long been hypothesised that ME/CFS may have an infectious cause; however, despite multiple investigations, a consistent correlation between ME/CFS and an infectious agent has not been found [2–5].

Metagenomics provides a new avenue to investigate microbial causes for diseases of unknown aetiology. Combined with next-generation sequencing, a metagenomic approach enables researchers to sequence all of the nucleic acid in a sample in order to examine its microbial content. Compared to targeted discovery approaches looking for specific microbes, metagenomics offers the distinct advantage of discovering unexpected microbial associations [6].

Despite its value as a holistic approach for investigating disease causation, metagenomics is not without its difficulties. Multiple studies have demonstrated contamination in metagenomic experiments from both environmental and reagent sources [7–9], meaning that any metagenomics experiment will likely reveal the presence of certain contaminant microbes. It is therefore vital to include a positive and negative control in any metagenomics study, with the positive control confirming that the method is able to detect microbes in the sample, and the negative control revealing those microbial sequences introduced through contamination. It is also vital to control for batch effects, which may occur when different samples are processed at different times. These problems may be exacerbated if samples with low microbial concentration are used, since the contaminants will occupy a relatively larger proportion of the total microbes present.

Despite these limitations, we chose to use a metagenomic approach to investigate potential microbial associations with ME/CFS, as we believe this to be the most sensitive and unbiased approach currently available. We also investigated a second disease of unknown aetiology, namely alternatively diagnosed chronic Lyme syndrome (ADCLS). ADCLS has a similar phenotype to ME/CFS, but is diagnosed on clinical grounds using alternative tests, the validity of which is questioned by major reference laboratories and the CDC [10, 11]. We performed a metagenomics case-control study comparing individuals with these two syndromes to healthy controls and a second set of controls with systemic lupus erythematosus (SLE), a chronic disease with accepted diagnostic criteria [12] whose sufferers also frequently experience fatigue. We chose to investigate plasma as this sample type was readily obtainable from both control and case patients and we hypothesized that there would be fewer confounding microbes in plasma compared to more complex sample types such as faeces, making etiologic associations easier to identify.

## Materials and Methods

### Recruitment

Recruitment was performed as detailed in Patrick et al. 2015 [11]. In short, participants were recruited from the general population in response to advertising or word of mouth. Participants with ME/CFS were required to meet the Canadian Case definition [13], as confirmed by their primary physician via a referral sheet (S1 Info) and reviewed by study coordinators. Patients were excluded if they were <19 years, unable to understand English, on antibiotic therapy in the last month, or diagnosed with another medical condition explaining their symptoms such as multiple sclerosis, diabetes, arthritis, hepatitis, HIV, cancer or a previous history of cancer (S2 Info). A complete list of all prescription and non-prescription medications taken by study participants is given in S1 Table. The protocol was approved by the University of British Columbia’s Clinical Research Ethics Board (H11-01998) Participants provided written informed consent, in a form that was approved by the UBC ethics board and adheres to the ethical conduct of human research subjects. Controls were age- and sex-matched to cases and any medical condition that precluded the diagnosis of ME/CFS also excluded eligibility as a control.

### Nucleic acid extraction and sequencing

Whole blood was drawn from participants into EDTA Vacutainer tubes (BD Biosciences, Mississauga, Ontario) and the plasma isolated and stored at -80°C until all collections were complete. Plasma specimen volumes ranging from 800 μL to 1 mL were then extracted using the NucliSens easyMAG system (bioMérieux, Craponne, France). A positive control consisting of a single patient respiratory specimen positive for human metapneumovirus (hMPV) by real time (RT)-PCR spiked into a pool of patient respiratory specimens negative for common respiratory viruses was extracted in parallel. Nuclease-free water (Promega Corporation, Fitchburg, WI) was included as a plasma-free negative control. All samples were eluted in 35 μL of elution buffer (bioMerieux). For each sample and control, 22.4 μl of the extracted nucleic acid was treated with Turbo DNase (Ambion, Inc., Austin, TX) for 30 minutes at 37°C. Each reaction was terminated using 0.1 X volume of inactivation reagent (Ambion, Inc., Austin, TX), followed by 5 min incubation at room temperature, centrifugation at 10,000 g x 1.5 min, and finally supernatant containing RNA was transferred to a new tube.

An 8.5 μl aliquot of RNA was reverse transcribed with tagged random nonamers (5′-GTTTCCCACTGGAGGATA-N9-3′), and subsequently amplified as part of a modified two-step procedure, as previously described [14, 15]. Amplified complementary DNA products were purified with AMPure beads (Beckman Coulter, Inc.) and the 5’ primer sequence flanking the random nonamers (Sol-B primer, 5′-GTTTCCCACTGGAGGATA-3′) was digested with 4 U BpmI (New England BioLabs Inc., Ipswich, MA), as previously described [16]. Purified libraries were then end-repaired, followed by adapter ligation and multiplexing, using the NEBNext Ultra DNA Library Prep Kit (New England BioLabs, Ipswich, MA). Insert sizes of 300 to 400 bp were targeted in the size selection process incorporated in the NEBNext protocol, and an additional AMPure bead treatment performed to minimize the presence of adapter dimers. To further decrease the adapter dimer content in the final batch of 32 samples, a modified gel size selection approach targeting fragments between 300 and 500 bp using Ranger Technology (Coastal Genomics Inc., Burnaby, BC) was performed following library preparation [17].

Sequencing was performed on an Illumina HiSeq 2000 sequencing system at McGill University and Genome Quebec Innovation Centre with 100 bp paired-end output (Illumina, Inc., San Diego, CA, USA). Nucleic acid concentrations were evaluated throughout the process using Qubit DNA and RNA High Sensitivity assay kits in a Qubit 2.0 fluorometer (Life Technologies, Carlsbad, CA).

To confirm the presence of bacteria identified by metagenomics, we performed conventional PCR amplification of the V3-V4 regions from the 16S rRNA-genes on the nucleic acid extracts prior to the DNase treatment and random amplification step. Each PCR reaction consisted of 1.5-mM MgCl_2_, 0.2-mM nucleotides, 0.4 μM of primers, 1.25 U of Hot Start Polymerase (Promega Corporation, Fitchburg, WI), 1 μl of template total nucleic acids, and water in a 25 μl volume. Thermocycler conditions were as follows: 94°C × 5 min, 35 cycles of 45 s at 94°C, 45 s at 50°C, and 60 s at 72°C, and a final cycle of 10 min at 72°C. Nuclease-free water (Promega Corporation, Fitchburg, WI) and *E*. *coli* genomic DNA were used as a negative and positive control, respectively. Aliquots of 5 μl for each PCR reaction were analyzed on a 1.5% agarose gel.

### Bioinformatics methods

Sequence files were downloaded as de-multiplexed BAM files and converted to fastq format using picard-tools-1.74 (http://picard.sourceforge.net). Illumina adapters and remaining Sol-B primer sequences from library preparation were removed using cutadapt v1.3 in paired end mode, with minimum length 70 bp [18]. Further filtering was then performed using prinseq-lite v0.20.2 for low complexity and paired-end duplicate removal with parameters: -lc_method dust -lc_threshold 7 -derep 14 [19], followed by a bespoke python script to further remove any reads which failed the Ilumina Chastity filter, had more than 20 bases in a row of the same call, contained at least one “N”, had less than 2/3 of the bases with a quality of > = 30 in the first half, or still contained the Sol-B primer, whilst also removing the B-tail and any reads less than 70 bp long.

Host subtraction was performed using bowtie2 v2.1.0 –very-sensitive [20] against a modified human reference consisting of the hg19 human reference plus human mtRNA (ftp://ftp.ncbi.nlm.nih.gov/refseq/H_sapiens/H_sapiens/CHR_MT/) and human rRNA (ftp://ftp.ncbi.nlm.nih.gov/refseq/H_sapiens/H_sapiens/RNA/). After extracting the unmapped reads using samtools v0.1.19 [21] and bamtools [22], further rRNA was removed using BMTagger [23] to the LSU and SSU SILVA databases (http://www.arb-silva.de/). Finally, prinseq-lite was used in single end mode to remove any duplicate single reads, not accounting for read pairs. Bacterial taxonomies were identified from the filtered non-host reads using Kraken [24] v0.10.6 with default parameters against the NCBI RefSeq database (downloaded 2015-07-21). We chose to use Kraken for bacterial analysis because of its increased speed compared to BLASTn, as well as its high specificity. For viral analysis, the more sensitive BLASTn was used, but against a reduced size database, also to increase speed. Thus viral taxonomies were identified from the same filtered non-host reads using BLASTn megablast with parameters -best_hit_overhang 0.25 -best_hit_score_edge 0.05, against the NCBI viral database (downloaded 2015-07-29). The bacterial and viral results were both then entered into MEGAN5 [25] for taxonomic assignment at the phylum and genus levels.

### Statistical methods

Statistical analysis was performed using R version 3.2.4. Clustering and heat maps were drawn using heatmap.3, multivariable regression performed using generalised linear models, and plots drawn using ggplot2.

## Results

### Sequencing results

Twenty-five ME/CFS cases, 25 healthy controls, 13 ADCLS cases and 11 SLE controls were included in the final study. Demographic details of the participants recruited are given in Table 1. cDNA sequences were obtained from all 74 samples, as well seven plasma-free negative controls and one positive respiratory control spiked with hMPV. Seven ME/CFS, two ADCLS, nine healthy and two SLE samples were sequenced twice. All fastq files are available from the ENA database (accession number PRJEB14374).

**Table 1:** Demographic and clinical characteristics of the study cohort.

Group	Healthy (N = 25) (N (%) or Median (IQR))	SLE N = 11) (N (%) or Median (IQR))	CFS (N = 25) (N (%) or Median (IQR))	ADCLS (N = 13) (N (%) or Median (IQR))	*P* SLE vs. Healthy	*P* CFS vs. Healthy	*P* ADCLS vs. Healthy
**Male**	4 (16%)	0 (0%)	4 (16%)	3 (23%)	0.3	1	0.7
**Age (years)**	53 (30;69)	51 (29;75)	54 (34;67)	45 (18;71)	0.5	0.9	**0.02**
**Ethnicity**					0.08	**0.04**	0.4
**Aboriginal**	0 (0%)	0 (0%)	2 (8%)	0 (0%)			
**White**	20 (80%)	5 (45%)	23 (92%)	13 (100%)			
**Chinese**	3 (12%)	3 (27%)	0 (0%)	0 (0%)			
**Other**	2 (8%)	3 (27%)	0 (0%)	0 (0%)			
**Duration of illness**						** **	** **
**Acute onset (vs. gradual)**	NA	4 (36%)	13 (52%)	3 (23%)	NA	NA	NA
**Karnofsky**	100 (65–100)	90 (50–100)	60 (40–80)	65 (60–90)	**0.002**	**<0.0005**	**<0.0005**
**SF36 physical**	55.8 (41.8–63.5)	40.1 (30.5–56.5)	24.7 (15.8–41.7)	30.4 (10.3–53.2)	**0.001**	**<0.0005**	**<0.0005**
**SF36 mental**	55.4 (32.3–61.2)	50.8 (30.1–58.7)	47.8 (17.3–58.1)	46.7 (27.7–59.3)	0.1	**0.003**	**0.02**

P-values calculated with Fisher’s Exact Test for categorical variables or Wilcoxon rank sum test for continuous variables; SLE—Systemic lupus erythematosus; CFS—Chronic Fatigue Syndrome; ADCLS—Alternatively Diagnosed Chronic Lyme Syndrome; N/A—Not Applicable. Values are N (%) for categorical variables and median (range) for continuous variables.

Sequencing was performed in three batches, with RNA extracted and libraries prepared on three separate occasions. Across all batches of sequencing results, there was a mean of 16,855,866 paired end reads sequenced per sample, of which the majority were removed during quality filtering and host subtraction, leaving a mean of 282,525 reads for analysis. We identified a significant difference in number of reads produced between sequencing batches (*P*<0.0001, Kruskal-Wallis), therefore, read counts were normalised by the number of filtered reads prior to host subtraction. A summary of read statistics for each sample can be found in S2 Table.

### Bacterial taxonomic investigation

We initially tested our bacterial bioinformatics pipeline using a mock community consisting of eight genera, generated by Peabody et al. [26]. Our pipeline successfully identified all eight genera, in 7,274,457/7,416,836 (98%) of reads, thus indicating a sensitivity and specificity of 98% for bacterial analysis. Results of the mock community analysis are given in S3 Table.

**Fig 1:**
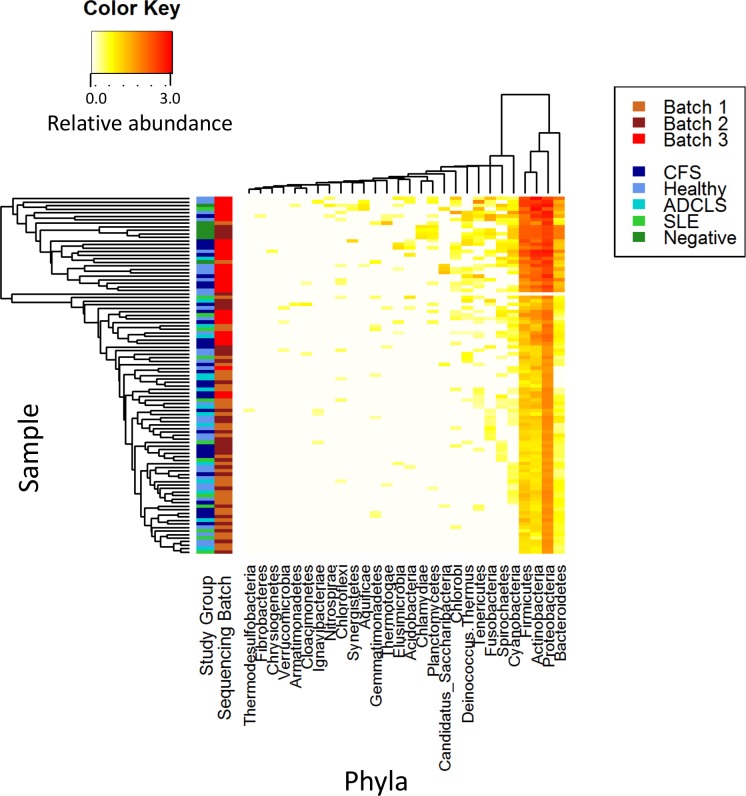
Heatmap of bacterial RNA present in each sample at the phylum level. Sample and phyla are ordered using hierarchical clustering. Samples, on the left of the plot, are coloured by study group and sequencing batch. Clustering demonstrates no relationship between group and phylum distribution. CFS = Chronic Fatigue Syndrome; ADCLS = alternatively diagnosed chronic Lyme syndrome; SLE = systemic lupus erythematosus.

Kraken identified the presence of RNA from 28 bacterial phyla and 512 bacterial genera in our samples, of which 20 (71.4%) phyla and 254 (49.6%) genera were present in one or more of the plasma-free negative control samples. RNA from Proteobacteria, Firmicutes, Actinobacteria and Bacteriodes were all detected at high abundance in both study samples and plasma-free negative controls (Fig 1).

In order to determine whether there were differences in RNA levels between samples, hierarchical clustering was performed. Hierarchical clustering provided no evidence of samples clustering according to study group, with the exception of the plasma-free negative control samples, which clustered together. However, clustering by sequencing batch was observed, with sequencing batch 3 largely separating out from the other two batches.

**Fig 2:**
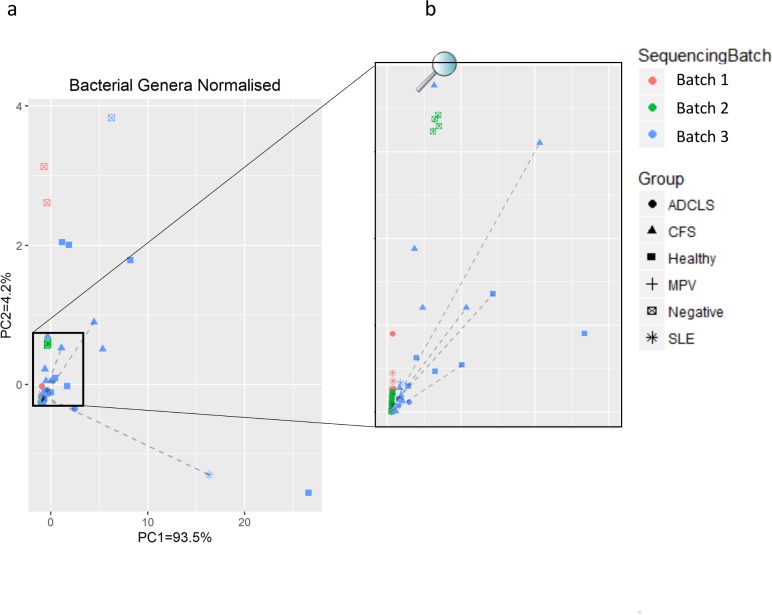
Principal Component Analysis (PCA) of normalised bacterial genera. Points are coloured by sequencing batch and shaped by study group. Dashed lines indicate the same sample sequenced twice. (a) complete PCA plot of all samples, (b) zoomed-in plot to highlight differences between batches.

To further investigate the relationship between bacterial RNA content and study group or sequencing batch, Principal Component Analysis (PCA) was performed at the phylum and genus levels using the normalised read abundances. Neither analysis showed samples from the same study group clustering together; however, samples did cluster according to the batch in which nucleic acid extraction and sequencing were performed (Fig 2).

To compare the effects of study group and sequencing batch on RNA content, we performed multivariable regression analysis on the raw reads, with duplicate samples randomly removed and without the plasma-free negative controls. After Bonferroni correction, required to reduce P values in order to correct for multiple comparisons [27], there were six genera whose level of RNA differed significantly between study groups: *Propionibacterium*, *Pandoraea*, *Acidovorax*, *Rubrivivax*, *Cupriavidus*, and *Alteromonas* (*P*<0.005), however, RNA corresponding to these six genera also differed significantly between sequencing batch. There were also 36 genera whose RNA differed significantly between sequencing batch only (*P*<0.005), indicating that sequencing batch had an effect on the RNA levels of more genera than study group (S4 Table).

Of the six genera whose RNA differed significantly between study group and sequencing batch, four (*Propionibacterium*, *Acidovorax*, *Cupriavidus* and *Alteromonas*) also had high levels of RNA in the plasma-free negative control samples and are known common contaminants [7, 28]. The remaining two (*Pandoraea* and *Rubrivivax*) did not differ significantly in RNA abundance between any single study groups using a Wald test to determine the effects of the individual groups [29], rather only at the group level.

Of note, Kraken analysis identified RNA from *Borrelia*, the bacterial genus that causes Lyme disease, in 16 samples, of which three were from participants with ADCLS. Further investigation of the reads matching *Borrelia* RNA revealed that only five mapped to *Borrelia* in both reads from the pair, of which only one pair was from a participant with ADCLS. Furthermore, using BLASTn, with the same parameters used for viral analysis against the NCBI nr database, revealed only one single read out of all 47 identified by Kraken matched to *Borrelia*, and this was from a healthy participant. Therefore, we were unable to find evidence for the presence of *Borrelia* in plasma from participants with ADCLS.

To validate the presence of bacteria identified through shotgun metagenomics, we employed PCR using universal 16S primers, binding to the V3-V4 regions of the 16S rRNA-genes, to see if we could amplify any bacterial 16S rRNA genes. We determined the limit of detection of this assay to be 24.1 molecules per μl. Using this method we were unable to amplify any 16S rRNA genes, even in the sample with the highest number of reads (0.5%) matching to RNA from bacterial phyla. This suggests that the level of bacteria is very low in our samples, and it is even possible that the observed bacteria came from contamination during sample processing. As the levels of bacteria were so low based on the PCR, results are highly subject to contamination and minor variances from extraction and other laboratory processes, highlighting the need for controls and tracking of reagents lots, as well as the batching of extraction, library preparation and sequencing runs.

### Viral taxonomic investigation

**Fig 3:**
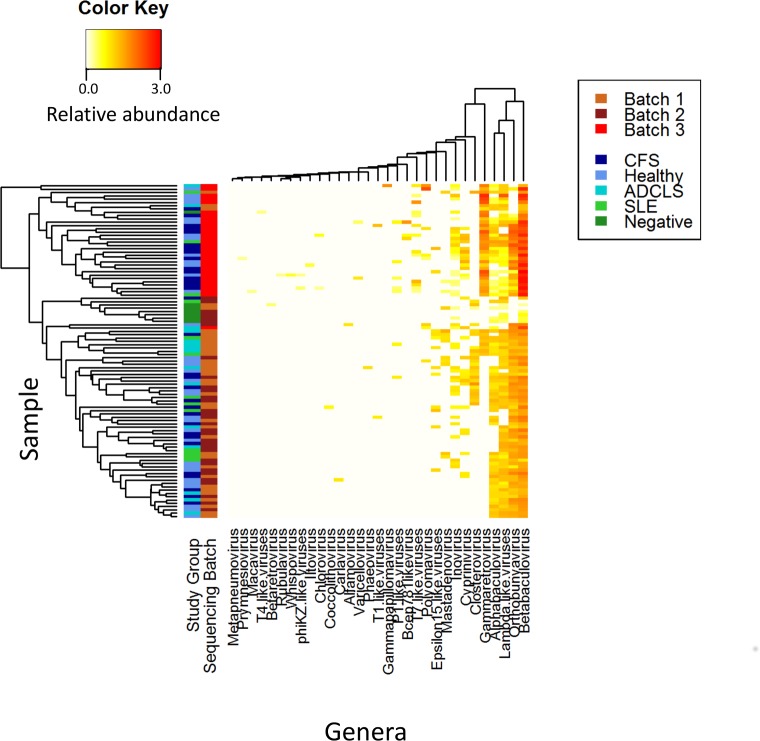
Heatmap of viral RNA present in each sample at the genus level. Samples and genera are ordered using hierarchical clustering. Samples, on the left of the plot, are coloured by study group and sequencing batch. Clustering demonstrates no relationship between group and phylum genus. CFS = Chronic Fatigue Syndrome; ADCLS = alternatively diagnosed chronic Lyme syndrome; SLE = systemic lupus erythematosus.

We also investigated the study samples for viral RNA content. Due to the comparatively small genome size of viruses compared to bacteria, we expected viral RNA to be present at lower abundance in our samples. Therefore, we used the more sensitive BLASTn megablast to map all filtered reads to the NCBI RefSeq viral database. RNA from six and 47 viral phyla and genera were identified respectively, of which RNA from three (50%) and 15 (31.9%) were in at least one plasma-free negative control sample (Fig 3). In the hMPV positive control 72/30,295 reads entered into BLASTn analysis against the viral database mapped to hMPV.

We again used multivariable regression to investigate the effect of study group and sequencing batch on viral RNA content, revealing that RNA from only one virus, *Betabaculovirus*, significantly differed between study groups after Bonferroni correction. However, despite reaching significance at the group level, no single study group differed from any other in its level of *Betabaculovirus* RNA using a Wald test. This virus also differed significantly between sequencing batch, with sequencing batch 3 having significantly higher abundance of *Betabaculovirus* RNA using a Wald test (*P*<0.0005). Such a significant difference between batches suggests that the batch effect was far larger than the study group effect. Furthermore, RNA from *Betabaculovirus* was present in the plasma-free negative control samples, particularly the one from batch 3, and since it is a dsDNA virus, we are measuring transcription rather than DNA copy number. Also, its natural hosts are arthropods, supporting our conclusion that it is a contaminant rather than a true component in human plasma. See S5 Table for complete results of the regression analysis.

## Discussion

Our metagenomic investigation of plasma RNA content revealed significant variation between sequencing batches, and potential sample contamination, but did not reveal differences in the plasma RNA content between individuals with ME/CFS or ACDLS and healthy controls or controls with SLE. Our findings highlight the necessary care that must be taken when performing metagenomic experiments both to control for batch-to-batch effects and the need to include negative and positive controls in the experimental design to identify contaminants.

We chose to analyse the RNA content of our samples, meaning that our results represent a composite picture combining the actual cellular abundance with metabolic and replicative activity of the microbes present. This therefore may not entirely reflect the bacterial abundance of each sample. Despite this limitation, batch-to-batch effects and sample contamination were both clearly identified, highlighting their strong impact. It should be noted that it is possible that differences between study groups would have been identified if we had performed DNA analysis, which is as a more precise measure of cellular abundance, however, the large batch and contamination effects, as well as the low levels of microbial RNA present, make this highly unlikely.

There has been little research performed on metagenomics of plasma. One study investigated the plasma microbiome in patients with HIV and found it was dominated by the orders Pseudomonadales, Lactobacillus, Burkholderiales, Bacillales and Enterobacteriales [30]. However, they did not use a negative control, meaning their results could be contaminants and indeed these orders were all found in the plasma-free negative controls used in our study.

In fact, it could be argued that a plasma microbiome does not exist at all in healthy subjects. Such an assertion is supported by the fact that even in the sample with the highest number of RNA reads mapping to bacteria in this study, we were unable to amplify any microbial ribosomal RNA from the plasma extracts. However, we believe it likely that the plasma has some microbial content, even if it is a low concentration of exogenous elements circulating in the blood that act as an indicator of microbial presence elsewhere in the body. As nucleic acid extraction techniques, purity of molecular biology reagents, and availability of methods that require less sample manipulation and amplification continue to improve, it will become possible to study nucleic acids from increasingly small volumes [31, 32]. Thus, future work may reveal a microbial component to human plasma that we are not yet able to identify.

Since all plasma-free negative controls used in this study were put through every step of our protocol, we are unable to determine where contamination arose. To determine the source of contamination, further experiments would be required, in which a new negative control was introduced at each step. Since our initial aim was not to measure contamination, our study did not use this approach. However, other studies have shown contamination arises from multiple sources, including ultrapure water [9] and nucleic acid extraction kits [7, 8].

It is also possible that some taxonomies identified could be false positives occurring due to incorrect alignments made by Kraken or BLASTn. Using a bacterial mock community, we calculated that our bioinformatics pipeline has sensitivity and specificity of 98%, suggesting that 2% of reads may be false positive or false negative alignments. This is particularly likely for bacterial genera seen at low levels, for example *Borrelia*, which was proven to consist of largely incorrect alignments when validated using BLASTn. Similarly, for viral alignments, *Orthobunyavirus* RNA was found in 84/102 (82%) of samples (data not shown), however, *Orthobunyavirus* is often identified due to non-specific alignments of bacterial sequences to viral databases, thus may not truly be present in our samples. It is likely that aligning all reads to the complete NCBI database using BLASTn would reduce false positive taxonomic assignments, however, such an analysis would be highly time consuming, hence alternative faster alignment methods were chosen.

The lack of a positive association between ME/CFS and ADCLS and plasma microbial content in this study does not mean that these syndromes do not have an infectious cause, and future experiments should examine the microbiome of other body tissues, including the cellular blood microbiome, which may harbour higher concentrations of microbes. The gut microbiome is of particular interest, as it has been associated with other chronic diseases, including for example, inflammatory bowel disease [33, 34], asthma [35], and multiple sclerosis [36]. In the ME/CFS field, 16S rRNA gene sequencing of stool samples from ME/CFS patients and healthy controls demonstrated a significant increase in *Lactonifactor* and decrease in *Holdemania* in ME/CFS patients compared to controls [37]. A second study also using 16S rRNA gene sequencing of stool samples found a significant decrease in *Actinobacteria* and one other phylum in ME/CFS patients compared to controls, but no significant differences in bacterial phyla from blood samples. However, both ME/CFS studies were small, with sample sizes of 43 and 10 ME/CFS patients respectively. Furthermore, neither study used a negative control, so they were unable to rule out contamination as a source of their differences. Thus, confirmation of these results requires replication in other populations and laboratories, with careful use of control samples.

Due to its exploratory nature, our study had a relatively small sample size (25 ME/CFS participants), which reduced our power to detect differences in the metagenome between study groups. A large sample size is particularly important for investigating ME/CFS, since it is a complex disease defined by a group of symptoms, which are not all required to be present. To reduce within-case heterogeneity, we chose to use the Canadian Case Definition for ME/CFS [13] as it was the most specific definition available at the time of study design; however, other definitions have since become available, such as the US Institute of Medicine case definition [38]. To further reduce heterogeneity between patients, some researchers have suggested dividing ME/CFS into subtypes, for example based on functional disability, speed of onset, or biomarkers [39, 40], which again may increase a study’s power to detect associations between groups. Subtyping would be highly useful in future studies, but was not practical with our sample size.

It is also important to perform case-control studies using carefully matched controls. In our study we matched for sex and age within five years. However, it may also be beneficial to match on other characteristics, such as ethnic background or level of activity, as ME/CFS sufferers often tend to be quite sedentary. Additionally, nested case-control studies may increase a study’s power to find significant associations without requiring a large number of ME/CFS participants.

Another possible method of matching is to follow the same ME/CFS patient over time. One of the hallmarks of ME/CFS is post-exertional malaise (PEM), which involves symptom flare-up after exertion and which can be simulated using an exercise test. Shukla et al. 2015 measured the blood and stool microbiome of the same patients before and after PEM and were able to find differences in the abundance of seven of the nine bacterial phyla they investigated [41].

## Conclusions

Our study was unable to find a positive association between the plasma RNA content of individuals with ME/CFS or ACDLS and healthy controls or controls with SLE. Plasma represents a difficult medium for metagenomic analysis, since very low levels of microbes mean it is very difficult to distinguish the microbiome from background contamination. This is not to say that associations with ME/CFS or other syndromes might not be identified in the microbiome of other body sites, or perhaps in the investigation of host factors such as host gene expression or immunological profiles. However, future exploration must be performed rigorously with large sample sizes, clear definitions and careful use of positive and negative controls.

## Supporting Information

S1 Info Physician referral sheet “Confirmation of diagnosis of CFS for Chronic Complex Diseases Study”. (DOCX)

S2 Info Inclusion and exclusion criteria for study. (DOCX)

S1 Table All prescription and non-prescription medications taken by study participants. (XLSX)

S2 Table Summary of read statistics per sample. (XLSX)

S3 Table Results of mock community analysis. Numbers are raw read counts.
(XLSX)

S4 Table Results of multivariable regression on bacterial genera, with study group and sequencing batch as independent variables, including mean and standard deviation of all bacterial genera by study group and sequencing batch. (XLSX)

S5 Table Results of multivariable regression on viral genera, with study group and sequencing batch as independent variables, including mean and standard deviation of all viral genera by study group and sequencing batch. (XLSX)
