# Supplementary material for: Metagenomic Investigation of Plasma in Individuals with ME/CFS Highlights the Importance of Technical Controls to Elucidate Contamination and Batch Effects
Source: PLoS One. 2016 Nov 2;11(11):e0165691. doi: 10.1371/journal.pone.0165691 (PMC5091812; doi:10.1371/journal.pone.0165691)
Supplement: S1 Info — (DOCX) [file pone.0165691.s001.docx]

**Confirmation of Diagnosis of CFS for Chronic Complex Diseases Study**

**This is to confirm that** __________________________________________(patient name) has been diagnosed with chronic fatigue syndrome according to the Complete Canadian Clinical Working Case definition as outlined below

1. **Fatigue:** Patient must have a significant degree of new onset, unexplained, persistent or recurrent physical and mental fatigue that substantially reduces activity level.
2. **Post-Exertional Malaise and Fatigue:** There is an inappropriate loss of physical and mental stamina, rapid muscular and cognitive fatigability, post-exertional fatigue and/or malaise and/or pain and a tendency for other associated symptoms within the patient’s cluster to worsen. There is a pathological slow recovery period – usually 24 hours or longer.
3. **Sleep Dysfunction:** There is un-refreshed sleep or sleep quantity or rhythm disturbance such as reversed or chaotic diurnal sleep rhythm.
4. **Pain:** There is a significant degree of myalgia. Pain can be experienced in the muscles and joints and is often migratory in nature. Often there are significant headaches of new type, pattern or severity.
5. **Neurological/Cognitive Manifestations:** Two or more of the following difficulties should be present: confusion, impairment of concentration and short-term memory consolidation, disorientation, difficulty with information processing, categorizing and word retrieval, and perceptual and sensory disturbances-e.g., spatial instability, and inability to focus vision. Ataxia, muscle weakness and fasciculations are common. There may be overload phenomena: cognitive, sensory-e.g., photophobia and hypersensitivity to noise-and/or emotional overload, which may lead to “crash” (temporary period of immobilizing physical and/or mental fatigue) periods and/or anxiety.
6. **At Least One Symptom** **from two of the following categories**:

**Autonomic Manifestations:** orthostatic intolerance-NMH, POTS, delayed postural

hypotension, vertigo; light-headedness, extreme pallor; nausea and IBS; urinary frequency and bladder dysfunction; palpitations with or without cardiac arrhythmia; palpitations, and exertional dyspnea.

**Neuroendocrine Manifestations:** loss of thermostatic stability-subnormal body temperature and/or marked diurnal fluctuation, sweating episodes, recurrent feeling of feverishness and cold extremities; intolerance to heat and cold; marked weight change-anorexia or abnormal appetite; loss of adaptability and tolerance for stress, worsening of symptoms with stress and a slow recovery.

**Immune Manifestations:** tender lymph nodes, recurrent sore throat and flu-like symptoms, general malaise, new sensitivities to food, medications and/or chemicals.

1. **The illness persists for at least six months:** It usually has a distinct onset, although it may be gradual.

**Physician name: __________________________________________Date**____________

**Signature:**
