# Supplementary material for: Metagenomic Investigation of Plasma in Individuals with ME/CFS Highlights the Importance of Technical Controls to Elucidate Contamination and Batch Effects
Source: PLoS One. 2016 Nov 2;11(11):e0165691. doi: 10.1371/journal.pone.0165691 (PMC5091812; doi:10.1371/journal.pone.0165691)
Supplement: S2 Info — (DOCX) [file pone.0165691.s002.docx]

**INCLUSION AND EXCLUSION CRITERIA FOR CCD STUDY**

| **Inclusion criteria (must answer YES to all questions)** | | **Yes** | **No** |
| --- | --- | --- | --- |
| 1 | Has the participant experienced symptoms of fatigue for at least six months at any point in the disease process?  (For healthy participants just answer yes to this question) |  |  |
| 2 | Does the participant fit the case definition for Chronic Fatigue Syndrome, Systemic Lupus, Lyme Disease or a healthy participant? |  |  |
| 3 | Does the participant understand English? |  |  |
| 4 | Is the subject 19 years old or older? |  |  |
| 5 | Did the subject sign the consent form? |  |  |
| **Exclusion criteria (must answer NO to all questions)** | | **Yes** | **No** |
| 1 | Has the patient been on antibiotic treatment in the past month? |  |  |
| 2 | Does the participant have any other diagnosed medical condition that fully explains the main symptoms of fatigue, sleep disturbance, pain, and cognitive dysfunction, such as Addison's disease; Cushing's Syndrome; hypothyroidism; hyperthyroidism; iron deficiency; other treatable forms of anemia; iron overload syndrome; diabetes mellitus; cancer; treatable sleep disorders such as upper airway resistance syndrome and obstructive or central sleep apnea; rheumatological disorders such as rheumatoid arthritis, lupus, polymyositis and polymyalgia rheumatica; immune disorders such as AIDS; neurological disorders such as multiple sclerosis (MS), Parkinsonism, myasthenia gravis and B12 deficiency; infectious diseases such as tuberculosis, chronic hepatitis, Lyme disease, etc.; primary psychiatric disorders and substance abuse. (Carruthers BM. Definitions and aetiology of myalgic encephalomyelitis: how the Canadian consensus clinical definition of myalgic encephalomyelitis works. J.Clin.Pathol. 2007;60(2):117-9)   |  |  |

Screen #: ______________________________________________

Form completed by: ______________________________________

Date: __________________________________________________
